# HIV-1 drug-resistance and drug-dependence

**DOI:** 10.1186/1742-4690-4-78

**Published:** 2007-10-25

**Authors:** Chris Baldwin, Ben Berkhout

**Affiliations:** 1 Laboratory of Experimental Virology, Department of Medical Microbiology, Center for Infection and Immunity Amsterdam (CINIMA), Academic Medical Center of the University of Amsterdam, the Netherlands

## Abstract

In this review, we will describe several recent HIV-1 studies in which a drug-dependent virus variant was selected. A common evolutionary route to the drug-dependence phenotype is proposed. First, the selection of a drug-resistance mutation that also affects the function of the targeted viral protein. Second, a compensatory mutation that repairs the protein function, but in the presence of the drug, which becomes an intrinsic part of the mechanism. The clinical relevance of drug-dependent HIV-1 variants is also discussed.

## Introduction to the HIV-1 drug-dependence phenomenon

We previously described the emergence of a drug-dependent HIV-1 variant in a patient on T20 (enfuvirtide) therapy [1]. This variant first acquired a resistance mutation in the T20-binding site of the envelope (Env) protein that provided resistance to the inhibitor, but at a fitness cost. The virus then evolved further to repair this fitness defect by introducing a second-site compensatory mutation in the Env protein. This evolution event took place in the presence of the inhibitor, which became critically involved in the mechanism of Env-mediated membrane fusion. This resulted in a virus variant with improved fitness that was both resistant and critically dependent on the inhibitor for its replication.

There have been several in vitro reports on the selection of partially and fully drug-dependent HIV-1 variants to a number of antiviral compounds that target different steps in the virus life cycle. We will argue that, in many cases, the evolution of the drug-dependence phenomenon occurs via a similar path: the selection of initial drug-resistance mutations that reduce the fitness of the virus, and subsequently the introduction of second-site compensatory mutations that evolve in the presence of inhibitor to improve the fitness of the virus. This scenario may result in a better replicating virus variant that mechanistically uses the inhibitor and such a variant will show severely reduced fitness when the inhibitor is removed from the environment.

The evolution of drug-dependence may depend on the mechanistic nature of the inhibitor. For example, inhibitors that mimic a certain sequence or domain of the virus such as the fusion inhibitor T20 may be more prone to select for drug-dependent viruses as the mimicking peptide is able to become involved in the mechanistic process of Env-mediated membrane fusion. As discussed in more detail below, protease resistant HIV-1 variants could also adapt and optimize protease activity in the presence of a protease inhibitor [2], but no such phenomenon has been reported thusfar for reverse transcriptase inhibitors. We will review all studies that report drug-enhanced or drug-dependent HIV-1 variants. There is a growing body of evidence to suggest that drug-dependence is a more common phenomenon. In many cases, drug-dependence will be missed because the virus does not replicate without the drug, which is usually an indication for the researcher to stop any further experimentation. Furthermore, a drug-dependence phenotype will easily be missed in the diagnostic resistance screening assays used today such as the MTT assay.

## HIV-1 entry and the T20-dependence phenotype

HIV-1 enters the human cell in 3 main steps: 1) attachment of the viral surface Env gp120 protein to the CD4 receptor on the target cells; 2) subsequent interaction of the Env-CD4 complex with a coreceptor, and 3) virus-cell membrane fusion mediated by the Env transmembrane gp41 protein. Changes within gp41 involve two leucine zipper-like motifs; heptad repeat 1 (HR1) and heptad repeat 2 (HR2) assembling into a highly stable six-helix bundle structure, which juxtaposes the viral and cellular membranes for the fusion event [3-5]. Peptide fusion inhibitors such as T20 can bind to one of the HR motifs and block this conformational switch, and thus inhibit viral entry [6-9].

**Figure 1:**
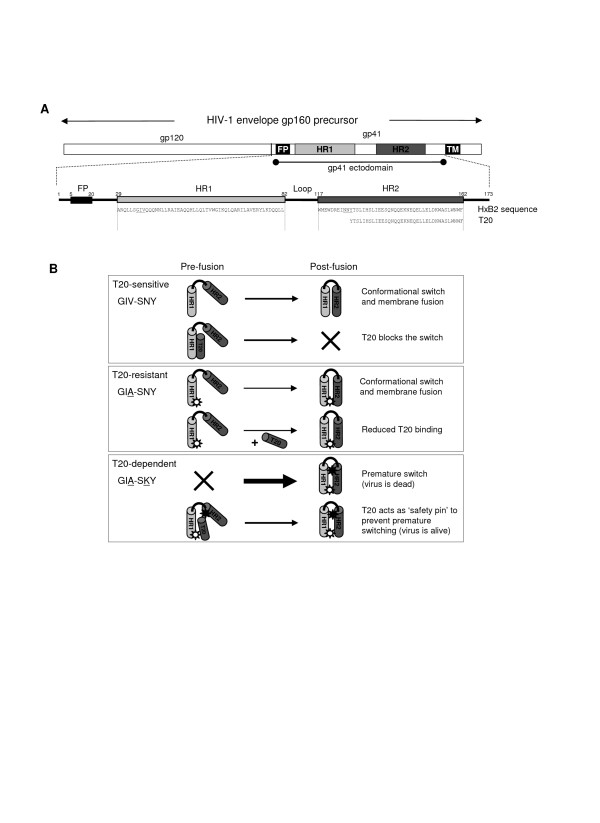
**(A) **Schematic of gp160, the gp120 and gp41 subunits and a close-up of the gp41 ectodomain. Indicated are the positions and amino acid residues of peptide based fusion inhibitor T20. The GIV sequence in HR1 (position 36–38) of gp41 is underlined. **(B) **Proposed model for T20-dependent viral entry. Each box depicts one of three scenarios: T20-sensitive (GIV-SNY), T20-resistant (GIA-SNY) and T20-dependent (GIA-SKY). A simplified gp41 ectodomain comprised of only one subunit of HR1 (light grey cylinder) and HR2 (dark grey cylinder) joined by a loop region (black line) is used to depict a pre-fusion and post-fusion state of the peptide. The thickness of the arrows represents the speed of the conformational switch between pre- and post-fusion conformations. A white star represents the GIA mutation in HR1 and a black star represents the SKY mutation in HR2. Explanations for each reaction are provided on the right hand side.

It is generally agreed upon that resistance to T20 is governed by changes in the HR1 region of gp41, specifically in a stretch of amino acids in and adjacent to the GIV motif (amino acids 36–45 of gp41) (Fig. 1A) [10]. There is accumulating evidence that other Env domains outside the HR1 domain also play a role. This role is either direct, e.g. in the formation of a fusogenic structure that is targeted by T20 and hence can modulate virus sensitivity to T20, or indirect by restoring Env function. One of these regions is the HR2 domain of gp41 that plays a crucial role in the formation of the 6-helix bundle as it folds in an anti-parallel fashion onto the pre-formed trimer of HR1 helixes.

In our report on the in vivo emergence of a T20-dependent virus [1], we described for the first time an HR2 amino acid change that was involved in T20-resistance. Briefly, we performed a genetic analysis of the entire HIV-1 gp41 ectodomain in the viral population from a patient that failed on T20 therapy. Sequence analysis revealed the acquisition of the known T20-resistance mutation GIA (GIV to GIA; mutated amino acid underlined) in HR1, but we also documented a subsequent change in a three amino acid SNY sequence of the HR2 domain (SNY to SKY). We demonstrated that the HR1-HR2 double mutant (GIA-SKY), which dominated the viral population after 32 weeks of therapy, was not only highly resistant to T20, but also critically dependent on the T20 peptide for its replication.

We proposed a mechanistic model that supports this novel feature of drug-dependent viral entry (Fig. 1B) [1]. Briefly, resistance to T20 is caused by the GIA mutation in HR1, which weakens the interaction with both T20 (resistance) and HR2 (gp41 6-helix bundle formation). The reduced HR1-HR2 affinity negatively impacts Env-mediated fusion and HIV-1 fitness [1,11]. The T20-dependence phenotype is caused by the SKY mutation in HR2, which stabilizes the HR1-HR2 interaction [1]. However, the SKY mutation creates a hyper-fusogenic Env-gp41 molecule that prematurely undergoes the conformational switch, which effectively kills virus infectivity. T20 is able to prevent this premature switch by preserving an earlier pre-fusion conformation, enabling gp41 to undergo the necessary conformational switch at the correct moment in the fusion process. The T20-control should be transient, as the peptide should leave the complex to allow the subsequent HR1-HR2 interaction.

We subsequently provided further evidence for this mechanistic model. First, according to this mechanistic model of T20-dependence, any compound that transiently interferes with the HR1-HR2 interaction should be able to support the replication of the T20-dependent virus. This critical test was performed with HR1- and HR2-targeting peptides and antibodies, and the results confirm the proposed mechanism (submitted for publication). The only exception was the T1249 fusion inhibitor, which acts as a dominant inhibitor because it does not leave the Env complex in time. This result indicates that the drug-dependence phenomenon can also be used in the preclinical testing of improved entry inhibitors, which should preferentially not stimulate the T20-dependent HIV-1 variant. Second, we used virus evolution to obtain insight into the T20-dependence mechanism [12]. Specifically, we allowed the T20-dependent virus to evolve in the absence of T20 to regain T20-independence. Escape variants with improved replication capacity appeared in 5 evolution cultures. Strikingly, 3 of these cultures selected the same amino acid change in the CD4 binding site of Env (glycine at position 431 substituted for arginine: G431R). This mutation was sufficient to abolish the T20-dependence phenotype by restoring viral replication in the absence of T20. Further experimentation indicated that the premature conformational switch is delayed by the second-site mutation in Env that affects the interaction with the CD4 receptor.

## How general is the T20-dependence phenotype: a common HR1-HR2 theme

Numerous clinical studies have reported T20-resistance mutations [1,10,11,13-20]. One clinical trial that enrolled 17 patients was used to track the evolution of sequence changes in HR1 and HR2 that are associated with T20-resistance [14]. Mutations in HR1 (amino acids 36–45) were noted in all patients. Isolates from 6 of 17 patients also developed the subsequent S138A change in HR2. It was proposed that the S138A mutation represents a compensatory mutation that increases T20-resistance, particularly when it co-exists with mutations at position 43 in HR1. Interestingly, careful analysis of the published results revealed a SIM-DKY variant in one of the patients after 24 weeks of therapy. This mutant resembles the T20-dependent GIA-SKY variant that we described [1]. However, little additional information is available, as not all mutations were tested in a molecular HIV-1 clone and analyzed for possible drug-resistance and drug-dependence. Studies that reported combined HR1/HR2 changes are summarized in Table 1.

**Table 1:** Combined HR1-HR2 mutations in the Env protein

**Inhibitor**	**gp41 mutations**		**Report**
T20	**HR1**	**HR2**	
T20	V38A (GIA)	N126K (SKY)	Baldwin *et al*, 2004 Ray *et al*, 2007
M87 (membrane anchored T20)	I48V	N126K	Hildinger *et al*, 2001
C34	I37K	N126K	Nameki *et al*, 2005
Retrocyclin RC-101	Q66R	N126K	Cole *et al*, 2006
T20	N43D	S138A	Xu *et al*, 2005 Perez-Alvarez *et al*, 2006 Ray *et al*, 2007
T20	N43D	E137K	Tolstrup *et al*, 2007

Another clinical trial study analyzed amino acid changes in the gp41 region of Env over a 40–72 week period in 4 patients that received T20 on top of an optimized antiviral regimen [13]. Three of the four patients initially developed T20-resistance mutations in the HR1 region and subsequently developed HR2 mutations. HR1 mutations occurred in the amino acid region 36–45 (G36D/E, N42T, N43D and L45M), whereas S138A was again the main mutation observed in HR2. Although they did not perform molecular recloning experiments, it can be concluded that compensatory changes in HR2 develop frequently within the course of T20 therapy.

Two very recent 2007 studies reported interesting compensatory changes in HR2 within the virus population of patients on T20 therapy [17,21]. The first study describes five treatment-experienced patients that were analyzed for Env sequences prior to T20 therapy and at the point of virologic failure [17]. The same double mutant that we reported [1], GIA-SKY, was isolated from one patient and confirmed to be highly resistant to T20. However, drug-dependence was not tested. In fact, all patients developed both HR1 and HR2 mutations, including the S138A change in HR2, which was seen in combination with the N43D mutation in HR1 in one patient.

In the second study, Env sequences were analyzed during the course of T20-therapy in 5 patients [21]. The N43D mutation in HR1 provided resistance to T20, but at a large fitness cost (92% decreased infectivity was measured). An interesting compensatory mutation in HR2 (E137K) restored the infectivity defect and further increased resistance to T20.

Thus, mutations in HR1 at residue 43 trigger a response in HR2 at residue 137 (E137K) or 138 (S138A). Interestingly, these HR1 and HR2 amino acid residues are juxtaposed in the post-fusion 6-helix bundle structure [14]. The introduction of N43D in HR1 introduces a negatively charged aspartic acid (D), which may be unfavorable in the formation of the 6-helix bundle as it approaches the negatively charged glutamic acid (E) at position 137. The compensatory HR2 mutation introduces a positively charged lysine (K) or a neutral charged alanine (A), which will avoid the repulsion and thus restore virus infectivity.

## T20-like drugs: a common HR1-HR2 theme

A novel gene therapy approach used a membrane-anchored gp41-derived peptide (M87) that includes the T20 sequence, which can protect cells from HIV-1 infection [22]. In an effort to characterize the mechanism of action of the membrane-anchored peptide in comparison to the soluble peptide T20, resistant HIV-1 variants were selected by serial virus passage using cells stably expressing the M87 peptide [23]. Sequence analysis of the resistant variants revealed the HR1 change I48V in combination with the HR2 change N126K, which is the same as the SKY mutation in the T20-dependent variant [1]. This double mutant was confirmed to be resistant to T20 but had a severe reduction in viral fitness in the absence of the T20 peptide.

Nameki *et al *[24] generated variants resistant to the C34 fusion inhibitor that has a similar mode of action as T20 [7,25]. A resistant variant with the I37K mutation in the GIV motif of HR1 and again the N126K mutation in the SNY motif in HR2 was reported. Binding assays revealed that the I37K mutation in HR1 impaired the binding of the C34 peptide, whereas the N126K mutation enhanced HR2 binding to the mutated HR1.

It is generally accepted that HR1 mutations cause resistance to T20/C34. The combined results indicate that HR2 mutations also play a major role in T20/C34-resistance development. HR2 changes may directly impact on the resistance phenotype, but are more likely to influence viral fitness because uncompensated HR1 mutations slow the fusion kinetics and reduce viral fitness [1,11]. Further studies should investigate the compensatory role of HR2 mutations on Env fusion kinetics and possibly drug-dependence.

## Other drug-dependencies in the Env protein

A 2006 report by Cole *et al *described the in vitro selection of resistant virus variants to retrocyclin RC-101 [26]. This drug is a cationic θ-defensin that inhibits HIV-1 entry by blocking 6-helix bundle formation in a similar manner to fusion inhibitors such as T20 and T1249 [27]. The resistant variants that emerged had mutations in HR1 and HR2 of gp41 as well as the CD4 binding domain of gp120 (C4 domain). It was noted that the HR1/HR2 double mutant, but also the HR1/HR2/C4 triple mutant, were not able to adequately infect cells in the absence of RC-101. Addition of RC-101 restored infectivity in a dose-dependent manner. Interestingly, the HR2 mutation is identical to the SKY (N126K) mutation that we reported in the T20-dependent virus [1].

Recently, a drug-enhancement phenotype was reported for an inhibitor-bound form of the CCR5 co-receptor [28]. HIV-1 infection can be inhibited by small molecules that target the CCR5 coreceptor and one of the most promising drugs is SCH-D (Vicriviroc) [29]. It was demonstrated that the fully SCH-D resistant viruses with mutations in the Env gene, enter target cells by recognition of the SCH-D bound form of CCR5. SCH-D does not inhibit these resistant viruses, and even enhances their infectivity modestly.

## Drug-enhancement of the HIV-1 protease

In 2003, Menzo *et al *described a partially drug-dependent phenomenon (drug-enhancement) when they reported that HIV-1 variants that are resistant to a protease inhibitor have enhanced fitness in the presence of the drug [2]. The drug-enhancement effect was associated with a large number of protease mutations and no single amino acid substitution that is responsible for this drug-enhancement could be identified. However, this report demonstrated that the virus could adapt and optimize protease activity in the presence of the inhibitor, which is of clinical significance as protease inhibitors are used extensively to treat HIV infected patients.

## Drug-dependence of the HIV-1 Gag protein

An in vitro study by Aberham *et al *in 1996 selected HIV-1 variants that are resistant to a non-immunosuppressive analog of cyclosporin A (CsA) [30]. The phenotype of all variants was not just drug-resistance, but full drug-dependence. The mutants selected in this study provided the first evidence that mutations in the Gag protein can confer resistance to CsA, and that these resistant variants were also critically dependent on CsA for their replication. Furthermore, the drug-dependent phenotype is very stringent, and only revertant viruses with the parental phenotype grew out in the absence of CsA. Subsequent reports proposed a mechanism of HIV-1 resistance to CsA [31,32]. Briefly, HIV-1 requires the incorporation of the peptidyl-prolyl isomerase cyclophilin A (CypA) into maturing virus particles via contact with the proline-rich domain of Capsid (CA) in the Gag polyprotein p55. Early findings on the involvement of CypA suggested that incorporation is necessary for the production of infectious virus particles [33,34]. More recent reports suggest that CypA protects HIV-1 CA from a restriction factor in human cells [35]. The mechanism will likely await identification of this putative restriction factor [36].

CsA binds to CypA and inhibits its incorporation into the virion particle. Resistance to CsA occurs when HIV-1 alters the proline-rich domain in CA to effectively become CypA-independent. Although the exact mechanism of CsA-dependence is not known, numerous models have been proposed [30-32,36]. Recently, a second-site compensatory mutation in a distal CA domain was selected that rescues the virus to a CsA-independent phenotype [36]. This study parallels our work on the evolution of a T20-independent variant [12].

In a recent 2006 study, Adamson *et al *reported a partial drug-dependence phenotype for HIV-1 variants that became resistant to the PA-457 (beviramat) inhibitor [37]. This drug blocks a late step in the Gag processing pathway, specifically the cleavage of SP1 from the C terminus of CA. Similar to our report on T20-dependence, they show that drug-resistant variants with a single resistance mutation had diminished replication capacity and second-site compensatory mutations were able to rescue virus replication. Thus, the first resistance mutation sets the stage for the second compensatory change that integrates the drug in the mechanistic process.

## Clinical implications of drug-dependent viruses

The evolution of drug-dependent HIV-1 variants has an obvious clinical relevance. The appearance of such variants during antiviral therapy may be an indication to modify the drug regimen. The switch to an alternative and effective drug regimen will obviously solve the problem, but we will discuss some other scenario's that specifically relate to the presence of a drug-dependent virus. Another problem is that the drug-dependence phenotype is easily overlooked in diagnostic drug-resistance tests. Improved screening assays would be of great advantage to patients as physicians could better define the therapy regimens. It is therefore important that current drug-resistance screening assays are modified to be able to detect the appearance of drug-dependent variants.

Should the treating clinician change the drug regimen when a drug-dependent HIV-1 variant is selected? One could consider stopping with the particular drug to which the virus has become dependent. However, the impact on the viral load will only be transient as archived drug-resistant and wild-type viruses will reappear quickly. Because the wild-type virus is likely to have a higher fitness (without drug) than the drug-dependent virus (with drug) it may in fact be better to continue treatment. An alternative approach would be to provide only sub-optimal amounts of the drug in question, which should lead to down-regulation of the viral load, yet prevent the reappearance of the wild-type virus. However, drug-resistant variants are likely to be favored in this context. Perhaps an alternating on/off treatment scenario provides a good treatment alternative. With drug, the drug-dependent virus will rapidly dominate the quasispecies. Without drug, the wild-type (and resistant) viruses will reappear. It is unclear if this on/off regimen is beneficial for the patient. Similar drug holidays are generally not advised, but the situation will be different with the presence of drug-dependent viruses.

An interesting difference between drug-resistant and drug-dependent viruses is at the level of the human population and virus transmission. Drug-resistant viruses are known to spread within the current epidemic [38], but this would seem impossible for T20-dependent viruses because the antiviral inducer drug is not available in the newly infected individual. The actual situation will differ for different drug-dependencies. Entry and RT inhibitor dependence will prevent the establishment of the integrated DNA provirus in the recipient. Drug-dependence that acts at later steps (e.g. Protease drugs) will also block virus replication, but only after an initial DNA provirus integration. In general, drug-dependent viruses will not be able to spread in the population, which could be another reason to try to maintain such variants in patients with a high risk profile of infecting others.
